# Frequency of occurrence and habitat selection shape the spatial variation in the antibiotic resistome in riverine ecosystems in eastern China

**DOI:** 10.1186/s40793-022-00447-9

**Published:** 2022-11-02

**Authors:** Chunxia Jiang, Haiyang Chen, Hans-Peter Grossart, Quanfa Zhang, Robby Stoks, Yi Zhao, Feng Ju, Wenzhi Liu, Yuyi Yang

**Affiliations:** 1 Key Laboratory of Aquatic Botany and Watershed Ecology, Wuhan Botanical Garden, Chinese Academy of Sciences, Wuhan, 430074 China; 2 University of Chinese Academy of Sciences, Beijing, 100049 China; 3 College of Water Sciences, Beijing Normal University, No 19, Xinjiekouwai Street, Beijing, 100875 China; 4 Leibniz-Institute of Freshwater Ecology and Inland Fisheries (IGB), 16775 Neuglobsow, Germany; 5 Institute of Biochemistry and Biology, University of Potsdam, Maulbeerallee 2, 14469 Potsdam, Germany; 6 Laboratory of Evolutionary Stress Ecology and Ecotoxicology, University of Leuven, B-3000, Leuven, Belgium; 7 School of Water Resources and Environment, China University of Geosciences, Beijing, 100080, China; 8 Key Laboratory of Coastal Environment and Resources of Zhejiang Province, School of Engineering, Westlake University, 18 Shilongshan Road, Hangzhou, 310024 Zhejiang China; 9 Danjiangkou Wetland Ecosystem Field Scientific Observation and Research Station, Chinese Academy of Sciences & Hubei Province, Wuhan, 430074, China

**Keywords:** Antibiotic resistance genes, Riverine ecosystem, Habitats, Anthropogenic impact

## Abstract

**Background:**

Riverine ecosystems are one of the most important reservoirs of antibiotic resistance genes (ARGs) in the environment, but the occurrence and controlling factors of ARG distribution in different habitats of riverine ecosystems remain poorly understood. In this study, a metagenomic approach was used to characterize ARG types and their abundance in different habitats (rhizosphere soil, surface bulk soil, bottom bulk soil, and sediment) of riverine ecosystems in eastern China. Sampling sites were located along different rivers of eastern China, which are geographically isolated. Differences in bacterial communities, mobile genetic elements (MGEs), pattern and intensity of human activities, climate, and other environmental factors at the sampling sites and habitats were expected to affect ARG occurrence.

**Results:**

ARGs were observed with high variations in diversity (44–206 subtypes) and abundance (6.85–105.68 ×/Gb). There were significant south-north differences in ARG occurrence in the same habitat, except for surface bulk soil. And the significant difference was found in ARGs among four southern habitats. South–north differences in ARGs of the same habitat were mainly attributed to the combination of different occurrence frequencies and habitat selections of ARGs. Differences in ARG profiles among the four habitats in the south and the north were both mainly attributed to the different occurrence frequencies of ARGs. Bacterial communities and MGEs (Mobile genetic elements) could account for the observed variance in the resistome of riverine ecosystems across eastern China. The co-occurrences of specific ARGs with bacterial communities and MGEs were more frequent at the northern sampling sites than in the south, and co-occurrence patterns (i.e. ARGs and bacterial communities or ARGs and MGEs) varied between the habitats. Moreover, building land in all habitats, except bulk soils, showed significant positive correlations with ARG abundance.

**Conclusion:**

This study reveals a high variance in the resistome of riverine ecosystems in eastern China and its controlling factors. We appeal to the importance of assessment of ARGs in the riverine ecosystem and the need for future prevention and intervention of ARG spread.

**Supplementary Information:**

The online version contains supplementary material available at 10.1186/s40793-022-00447-9.

## Introduction

Antibiotic resistance is increasingly recognized as a growing global public health threat, and without control, it is expected to cause 10 million deaths worldwide by 2050 [1, 2]. Many clinically relevant antibiotic resistance genes (ARGs) originate from the environmental resistome [3–5]. In addition, enrichment and transmission of ARGs in the environment can be influenced by microbial hosts and mobile genetic elements (MGEs, which mediate horizontal gene transfer), and other factors including anthropogenic activities, heavy metals, antibiotics pollution, and specific physicochemical factors [6–8]. A comprehensive understanding of the environmental resistome and its major drivers are urgently needed to dissect mechanisms controlling ARG occurrence in the environment. In the natural environment, soil and sediment are the two most important environmental reservoirs for diverse ARGs [4, 9].

The abundance and diversity of ARGs vary in soils and sediments of different ecosystems [6, 10, 11]. As one of the largest biodiversity reservoirs, riverine ecosystems participate in a variety of ecological processes and thus play an important role in human nutrition and well-being. However, riverine habitats, especially waters, are susceptible to anthropogenic pollution that is released through different sources, such as agricultural runoffs [12], sewage discharges [13], medical wastewater [14], and leaching from nearby farms [15], while riverine sediments are in close contact with river water and often act as reservoirs for pollutants including ARGs. Biological pollution (including ARGs) of rivers, an important freshwater resource, has become one of the most pressing problems for humankind. Singh et al. [10] showed that antibiotic resistance genes are commonly found in rivers across the globe, and antibiotic usage patterns can alter the trend of riverine resistance. In addition, it has been shown that MGEs [16], physicochemical factors (e.g., salinity, oxidation-reduction potential, pH) [11], and anthropogenic activity [17–19] can influence the ARG occurrence in rivers. It is clear that ARGs in river ecosystems are ubiquitous and susceptible to interference by their surroundings.

Heavy pollution loadings and insufficient hydrodynamics limit dispersal, dilution, and reoxygenation processes in the river, lowering the ecological state and thus reducing the self-purification capacity [20]. The southeastern area of the Hu Huanyong Line accounts for approximately 36% of the land area and is home to 96% of the total population of China [21]. This area is subject to more frequent anthropogenic activities, resulting in higher antibiotic emission densities in the southeastern than northwestern rivers [22]. It is well known that the trend of the total ARG abundances in environments closely matched well with anthropogenic disturbance and antibiotic selection pressures in the environments [18, 23]. Therefore, the rivers in the southeastern area of the Hu Huanyong Line (hereafter referred to as “eastern China”) may have serious ARG contamination, and this pollution is closely linked to public health. The riverine network in eastern China is complex, and there are marked differences in human activity patterns and climate from south to north (Qinling–Huaihe Line as the boundary) [21]. The study of ARGs in southern and northern rivers can contribute to understanding the differences in ARG occurrence and the mechanisms underlying their propagation in different habitats.

With the increasing availability of metagenomic data in public databases, a global view of the soil and sediment resistome is possible [7, 8, 24, 25]. While these studies provide a useful overview, they lack resolution on the effect of different environments and environmental drivers on ARG occurrence, which is crucial to understand microbial community assemblies in soils and sediments related to their resistome composition. While most contemporary studies of ARGs in river ecosystems are limited to riverine sediments or water bodies, riparian rhizosphere soil and bulk soil are not given due consideration.

To clarify the problem of ARG contamination in the riverine ecosystem of eastern China, sampling sites were set up on the north and south sides of the Qinling-Huaihe Line, and samples were collected from different habitats at 30 sampling sites, i.e. the rhizosphere, river bank, and channel. We addressed the following objectives: (i) geological distribution of ARG subtypes and abundance in the different habitats, (ii) identify differences and specific associations of ARGs in these habitats, and (iii) determine the interference of surroundings on the ARG dissemination in the riverine ecosystem of eastern China. This study improves our understanding of the occurrence and types of riverine ARGs and enables a better evaluation of public health risk caused by ARGs in the river environment.

## Materials and methods

### Field sampling and DNA extraction

**Fig. 1:**
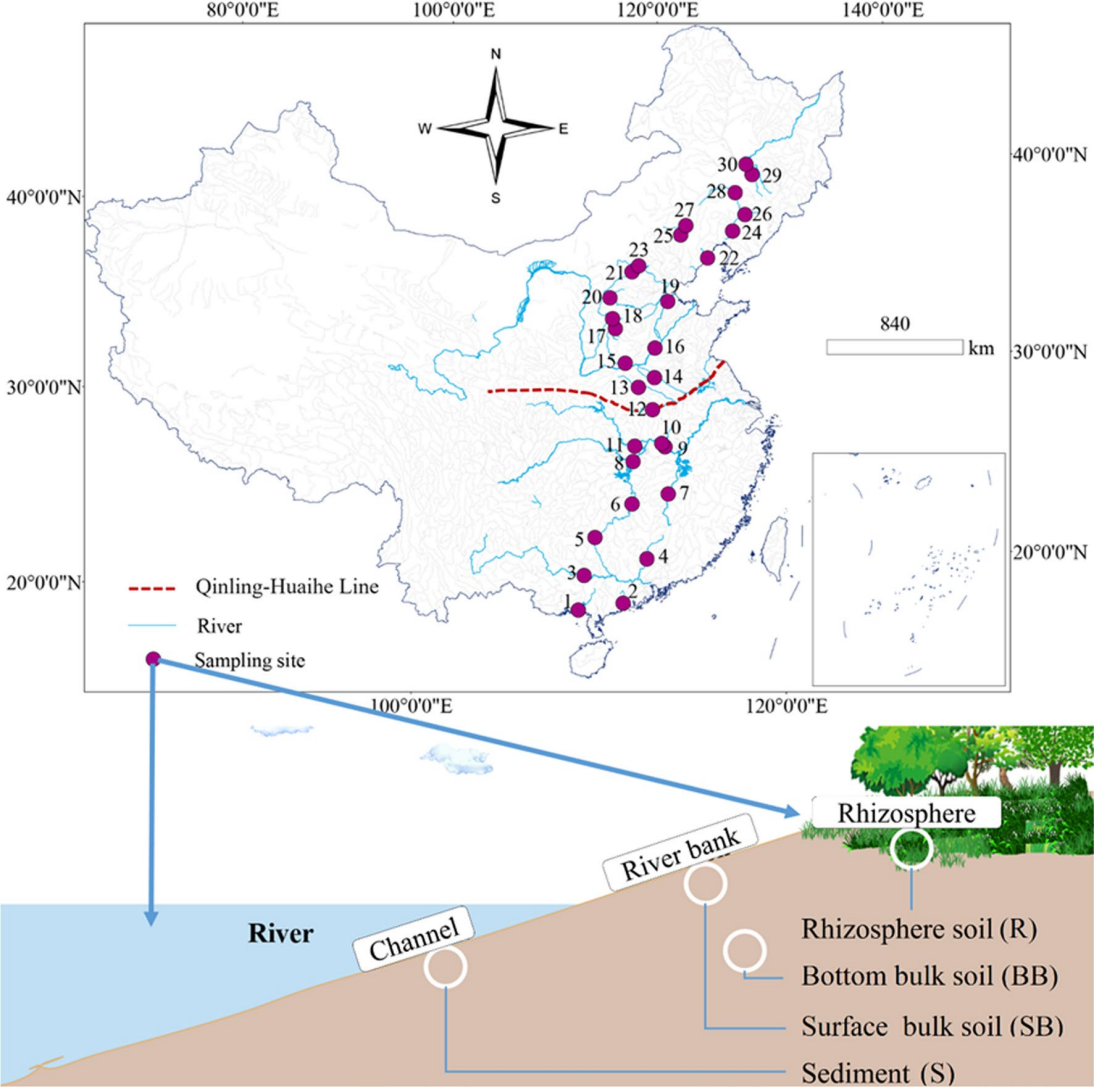
Sampling sites (upper plot) and habitats (lower plot) of the riverine ecosystem in eastern China. Rhizosphere soil (R) was collected from the rhizosphere; surface bulk soil (SB) was collected 0–20 cm below the bulk soil surface; bottom bulk soil (BB) was collected 40–60 cm below the bulk soil surface; sediment (S) was collected 0–20 cm below the sediment surface

In this survey, 30 sampling sites were selected from 30 rivers in eastern China and located near urban or rural areas (Fig. 1). At each sampling site, four habitats were considered: rhizosphere soil (0–20 cm), surface bulk soil (0–20 cm), bottom bulk soil (40–60 cm), and riverine sediment (0–20 cm). Simultaneously, two subsamples of each sample were taken, one was collected into a 50 mL sterile plastic centrifuge tube for molecular analyses, and the other was collected and preserved in a ziplock bag for analyses of physicochemical properties. The subsamples used for bioinformatic analyses were immediately transferred to − 80 °C for storage until further DNA extraction. The detailed information of samples is listed in the supplementary table (Additional file 1: Table S1). The genomic DNA was extracted using the PowerSoil^®^ DNA Isolation kit (MoBio, Carlsbad, California, USA) following the standard protocol and stored at − 80 °C for further molecular analyses.

### Metagenome sequencing and preprocessing of raw metagenomic reads

For library construction, the genomic DNA concentration was quantified using the ExKubit dsDNA HS test kit (ExCell Biotech Co., Ltd., Shanghai, China) with a Qubit fluorimeter (Invitrogen, Carlsbad, CA, USA). The confirmed high-quality genomic DNA was used for metagenome sequencing. Metagenomic sequencing libraries were generated using the TrueLib DNA Library Rapid Prep Kit for Illumina (Vazyme Biotech Co., Ltd., China) following the manufacturer^’^s recommendations, and index codes were added to attribute sequences to each sample. Then, sequencing was conducted using the NovaSeq 6000 System (Illumina, San Diego, CA, USA) (2 × 150 bp paired-end reads) with an insert size of 350 bp. Finally, approximately 6 Gb of raw data was obtained and further filtered to remove the adapter and low-quality reads, including reads with a N base content up to 10% of the read length and reads with low-quality bases (quality values ≤ 10) exceeding 50% of the read length.

### Metagenome data analyses

#### Metagenome assembly and gene prediction

Clean reads were assembled using MEGAHIT (v1.1.2), and the contigs longer than 500 bp were employed to predict open reading frames (ORFs) using MetaGeneMark (v3.25). The CD-HIT (v4.6.8) was used to cluster ORFs with 95% identity and a coverage > 90%, resulting in a non-redundant ORF set [7].

#### Identification of ARGs, MGEs, and taxonomic annotation

To annotate ARGs, MGEs and VFs, the predicted protein sequences of ORFs were used to align against the DeepARG [26] and the MGE database [27], respectively, using BLASTP implemented in DIAMOND software (v0.9.17.118) [28] with an e-value cutoff of 1e^− 10^. Sequences were annotated as ARG-, MRG- ORFs when the BLASTP alignment exceeded 60% of identity at query coverages of > 70%. For comparison, coverages of the identified ORFs were normalized to the size of each metagenome (times per Giga base, ×/Gb)) [29]. In addition, a metagenome classifier Kaiju (v1.6.3) was used for taxonomic annotation, in which reads were searched against the proGenomes database [30] for maximum exact matches at the protein level using Burrows-Wheeler transform. ARG-carrying contigs were also classified taxonomically using Kaiju (v1.6.3). The co-occurrence of ARGs and MGEs on contigs (Length < 5 kb) was obtained by collating the annotation results of the two sequence types.

### Climatic parameters, land use types, human population density, and physicochemical parameters

The longitude, latitude and altitude of sampling sites were recorded using a global positioning system (Unistrong, Beijing, China). Climatic factors, including mean annual temperature (MAT) and mean annual precipitation (MAP) of sampling sites, were obtained from the China Meteorological Data Sharing Service System (http://data.cma.cn/). The proportion of land use types (i.e., the proportion of cultivated land, building land, forest land, grassland, water area, and unutilized land) (hereinafter referred to as “cultivated land”, “building land”, “forest land”, “grassland”, “water area”, and “unutilized land”) and human population density at the sample sites were analysed using ArcGIS v 10.6.

Soil or sediment physicochemical properties including moisture, density, texture (i.e., the proportion of sand, silt, and clay) (hereinafter referred to as “sand”, “silt”, and “clay”), total carbon (TC), organic carbon (TOC), ammonium (NH_4_^+^), nitrate (NO_3_^–^), total nitrogen (TN), Ferrum (Fe), ferrous iron (Fe^2+^), available iron (AFe), total phosphorus (P) and sulfate (SO_4_^2−^) were determined in the laboratory, as described in detail in our previous work [31, 32]. Other parameters, including pH and electric conductivity (EC), were measured using a YSI Professional Plus multi-parameter water quality meter (YSI Inc., Yellow Springs, Ohio, USA).

### Statistical analyses

Herein, the four habitats (rhizosphere soil, surface bulk soil, bottom bulk soil, and sediment) were divided into eight specific habitats, using the south-north boundary “Qinling-Huaihe Line”. The eight habitats were southern rhizosphere soil (SR), southern surface bulk soil (SSB), southern bottom bulk soil (SBB), and southern sediment (SS), as well as northern rhizosphere soil (NR), northern surface bulk soil (NSB), northern bottom bulk soil (NBB), and northern sediment (NS).

Shared and unique ARGs harbored by different habitats were visualized by the bipartite network analyses using the Yifan Hu layout in Gephi. The significance of differences between habitats based on weighted Bray-Curtis distance matrice was determined by both non-metric multidimensional scaling (NMDS) and analyses of similarities (ANOSIM). Analyses of variance (ANOVA) was followed by Tukey’s test. The Source Tracker was conducted by “src/SourceTracker.r” in R [33].

Kruskal-Wallis tests were used to identify differential ARGs (ARGs with significant differences between/among habitats) for the studied habitats. Specificity-occupancy (SPEC-OCCU) plots were used to visualize the characteristic of differential ARGs. Specificity is defined by the proportion of mean ARG abundance in the samples of a habitat (H), and occupancy is defined as the relative frequency of ARG occurrence in the samples of H [34].

1 $${\text{Specificity}} = \frac{{{\text{N}} - {\text{individuals}}_{{ARG,H}} }}{{{\text{N}} - {\text{individuals}}_{{ARG}} }}$$

2 $${\text{Occupancy}} = \frac{{{\text{N}} - {\text{sites}}_{{ARG,H}} }}{{{\text{N}} - {\text{sites}}_{H} }}$$

N-individual_*ARG,H*_ is the mean number of individual ARGs across all samples of a habitat H, while N-individual_*ARG*_ is the sum of the mean number of individual ARG over all habitats; N-sites_*ARG,H*_ is the number of samples in H where a specific ARG is present, while N-sites_*H*_ is the total number of samples in H [35]. Specificity and occupancy were subsequently used as the axes in the SPEC-OCCU plots.

Mantel test was conducted to determine the correlation of two distance matrices. Redundant analyses (RDA) was subsequently conducted to elucidate the relationship between parameters and specific ARGs. Envfit function of the vegan R-package was used to select the parameters significantly related to ARGs in a habitat. A correlation between two variables (i.e. ARG and parameter) was considered statistically robust if the Spearman’s correlation coefficient (ρ) was > 0.8 and the P-value was < 0.01. To reduce the chances of obtaining false-positive results, the P values were adjusted with a multiple testing correction using the Benjamini-Hochberg method [36]. The robust pairwise correlations of any two specific items were used to construct their co-occurrence networks. Network analyses were performed in the R environment using the vegan, igraph and hmisc packages, and the visualization was conducted in Gephi 0.9.2 software using the Fruchterman Reingold layout [37].

## Results

### Overall occurrence of ARGs in the riverine ecosystem of eastern China

In total, 469 ARG subtypes belonging to 24 ARG types were detected in the studied river ecosystems of eastern China (Additional file 2: Fig. S1 (a)). The number of ARG subtypes detected in the rhizosphere soil, surface bulk soil, bottom bulk soil, and sediment were 384, 331, 317, and 360, respectively. The predominant ARG consisted of multidrug resistance genes with 125 subtypes accounting for 26.7% of the total number of ARGs, followed by beta-lactam resistance genes with 103 subtypes detected (22.0%) (Additional file 2: Fig. S2 (a)).

The total relative abundance of ARGs varied from 6.85 to 105.68 per Gb, with an average of 39.33 per Gb (2: Fig. S1 (b)). The relative ARG abundance detected in rhizosphere soil, surface bulk soil, bottom bulk soil, and sediment were 42.45, 37.13, 39.64, and 38.32 per Gb, respectively. The most dominant ARG type consisted of multidrug resistance genes accounting for 32.82% of the total relative ARG abundance, followed by macrolides-lincosamides-streptogramins (MLS) resistance genes (20.2%) ((Additional file 2: Fig. S2 (b)). The results revealed that six multidrug resistance genes (*acr*B, *bep*E, *mex*F, *mex*W, *msb*A, and *sav1866*), one trimethoprim resistance gene (*dfr*E), one mupirocin resistance gene (*ile*S1), one MLS resistance gene (*mac*B), and one quinolone resistance gene (*mfd*) were ubiquitous in all habitats with a 100% detection rate (i.e., core ARGs); among them, gene *mac*B had the highest abundance (0.95–11.93 per Gb) ((Additional file 2: Fig. S3). In addition, polymyxin resistance gene *mcr*-1 was found in very low abundance in a few samples.

**Fig. 2:**
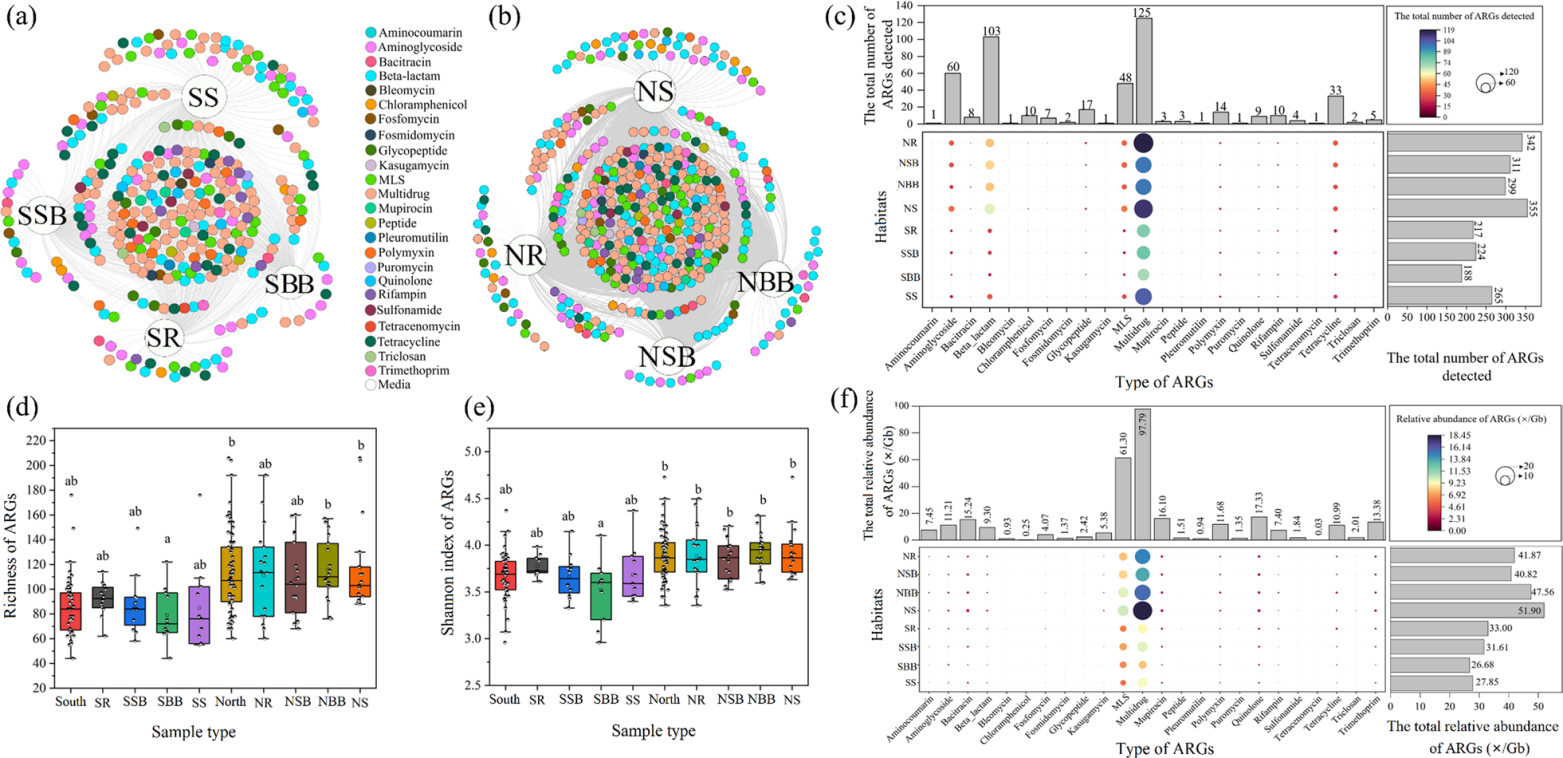
Occurrence and diversity of ARGs in rivers of eastern China. Bipartite association networks show unique and shared ARGs of different habitats in the south (**a**) and north (**b**), respectively. Number (**c**) and relative abundance (**f**) of ARGs detected in the different habitats. d, e richness (**d**) and Shannon index (**e**) of ARGs in different habitats. SR and NR represent rhizosphere soil in the south and north, respectively; SSB and NSB represent surface bulk soil (0–20 cm below bulk soil surface) in the south and north, respectively; SBB and NBB represent bottom bulk soil (40–60 cm below bulk soil surface) in the south and north, respectively; SS and NS represent sediment (0–20 cm below sediment surface) in the south and north, respectively. MLS represent macrolides-lincosamides-streptogramines

Alpha diversity values of ARGs are given in (Additional file 1: Table S2. The Shannon index of ARGs was 3.83, 3.78, 3.77 and 3.84 in rhizosphere soil, surface bulk soil, bottom bulk soil and sediment, respectively. Further observations revealed that both ARG number and relative abundance varied differently in the south and north when separated by the Qinling-Huaihe Line (hereinafter referred to as “south” and “north”). ARG number and relative abundance in the four habitats showed little variations in the south and large variations in the north (Additional file 2: Fig. S4). Both ARG richness and Shannon index were lower in the south than in the north, though they were not statistically significant (Fig. 2d, e).

### Cross-habitat comparison of richness and relative abundance of ARGs

To dissect the differences in ARG distribution in the riverine ecosystems of eastern China, ARG occurrence in the eight specific habitats (i.e. SR, NR, SSB, NSB, SBB, NBB, SS, and NS) were further analysed (Fig. 2). The bipartite association network showed that, in the south and north, the number of unique ARGs in different habitats was low and most ARGs occurred in all habitats (shared ARGs) (Fig. 2a, b). Multidrug resistance genes predominated in the shared ARGs. In both the south and north, the highest numbers of unique ARGs were found in the sediment (SS/NS), followed by rhizosphere soil (SR/NR). The total number and abundance of ARGs revealed a similar trend, with the highest values occurring in NS and the lowest values in SSB (Fig. 2c, f). Multidrug resistance genes accounted for the highest proportion in both number and abundance (Additional file 2: Fig. S5). ARG number and abundance were higher in all northern habitats than in the south (Fig. 2c, f), though there were no significant differences in ARG diversity between north and south (Fig. 2d, e).

**Fig. 3:**
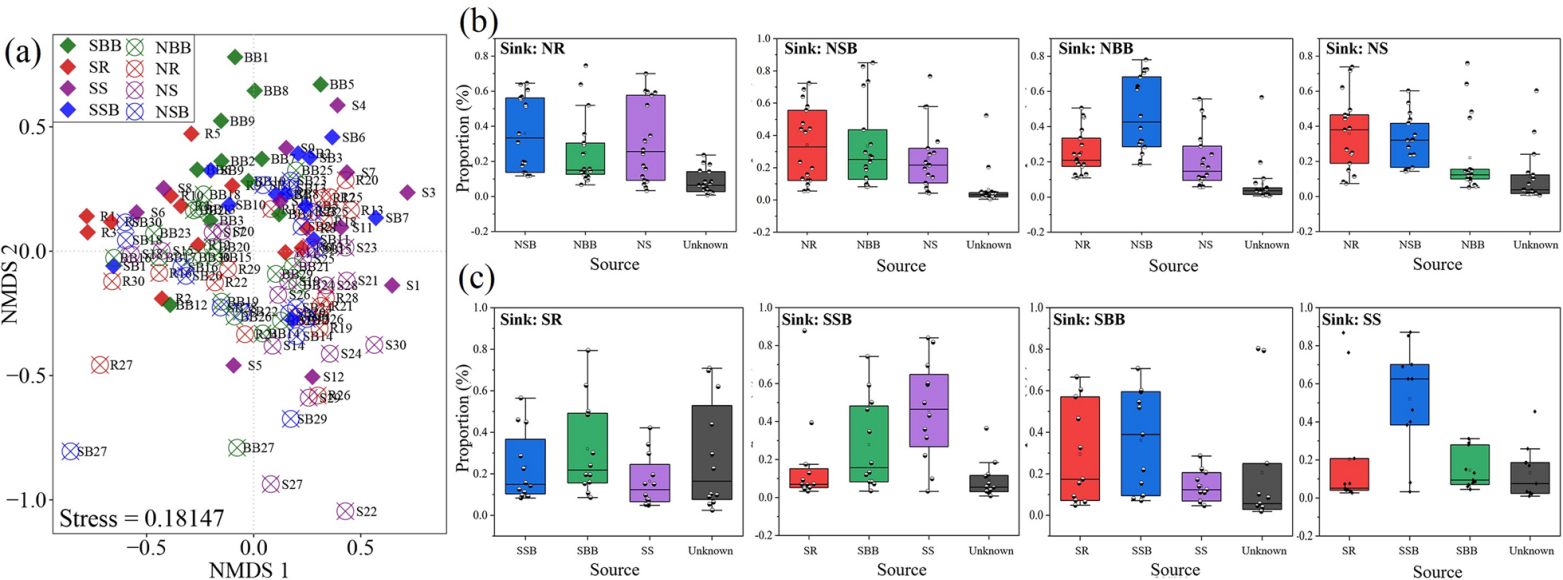
Differences and associations of ARGs in Eastern Chinese riverine ecosystem. **a** Non-metric multidimensional scaling (NMDS) analysis showing resistome profiles of soil and sediment samples of the different habitats. The percentage of ARGs in a specific sample habitat associated with all other sample habitats in the north (**b**) or south (**c**). (For abbreviations, see legend of Figs. 1 and 2)

To further reveal differences in ARGs in the riverine ecosystems of eastern China, NMDS analysis was performed to show differences in ARGs among different habitats (Fig. 3a). ANOSIM further confirmed that ARGs in the south were significantly different from those in the north (North vs. South, *P* = 0.001). Except for the surface bulk soil, there were significant differences in habitat-specific ARGs (i.e. rhizosphere soil (*P* = 0.035), bottom bulk soil (*P* = 0.002), and sediment (*P* = 0.003)) between south and north. There was a significant difference in ARGs between the four southern habitats (*P* = 0.011).

Source Tracker analysis showed the association of ARGs among the habitats in the south (SR, SSB, SBB, SS) (Fig. 3c) and the north (NR, NSB, NBB, NS) (Fig. 3b). Our results show that ARG sources differed for the same habitat (rhizosphere soil, surface bulk soil, bottom bulk soil, and sediment) in the south vs. the north, except for the bottom bulk soil. Although ANOSIM results showed no significant difference between surface bulk soil ARGs in the south and north (SSB vs. NSB), Source Tracker found that ARG sources in the surface bulk soil in the south (SSB) and north (NSB) were diametrically opposed (SSB: rhizosphere soil < bottom bulk soil < sediment, NSB: sediment < bottom bulk soil < rhizosphere soil). The numerical similarity in the mutual contribution of ARGs across northern habitats provided validation for non-significant differences in ARGs across northern habitats. In contrast, the mutual contribution of ARGs in the southern habitats fluctuated more, which is consistent with significant differences in ARG distribution among the southern habitats.

### Occupancy and specificity of differential ARGs in the studied habitats

**Fig. 4:**
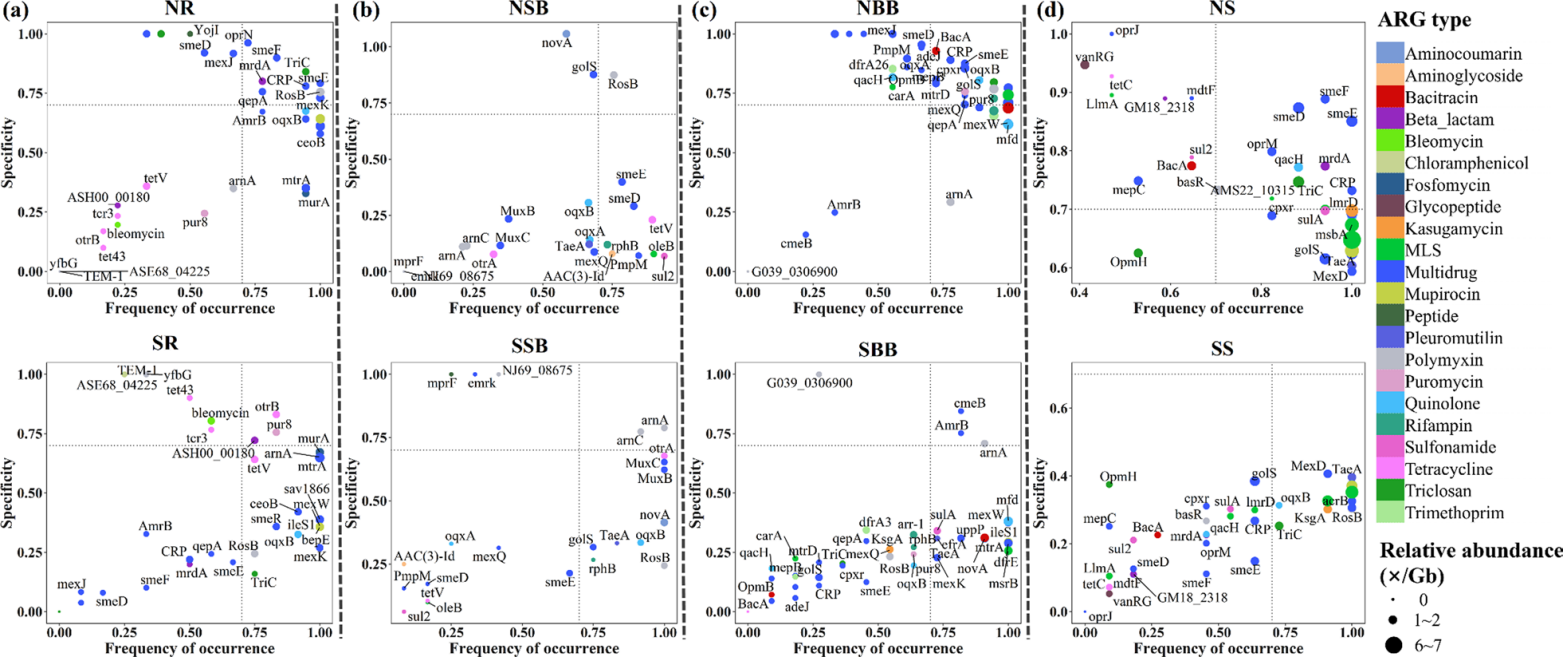
SPEC-OCCU plots showing ARGs that differ between two habitats in the south and north. **a** SR versus NR; **b** SSB versus NSB; **c** SBB versus NBB; **d** SS versus NS; the x-axis represents occupancy, i.e. how well an ARG is distributed in each habitat across all sites; the y-axis represents specificity, i.e. whether ARGs are also found in other habitats. (For abbreviations, see legend of Fig. 2)

**Fig. 5:**
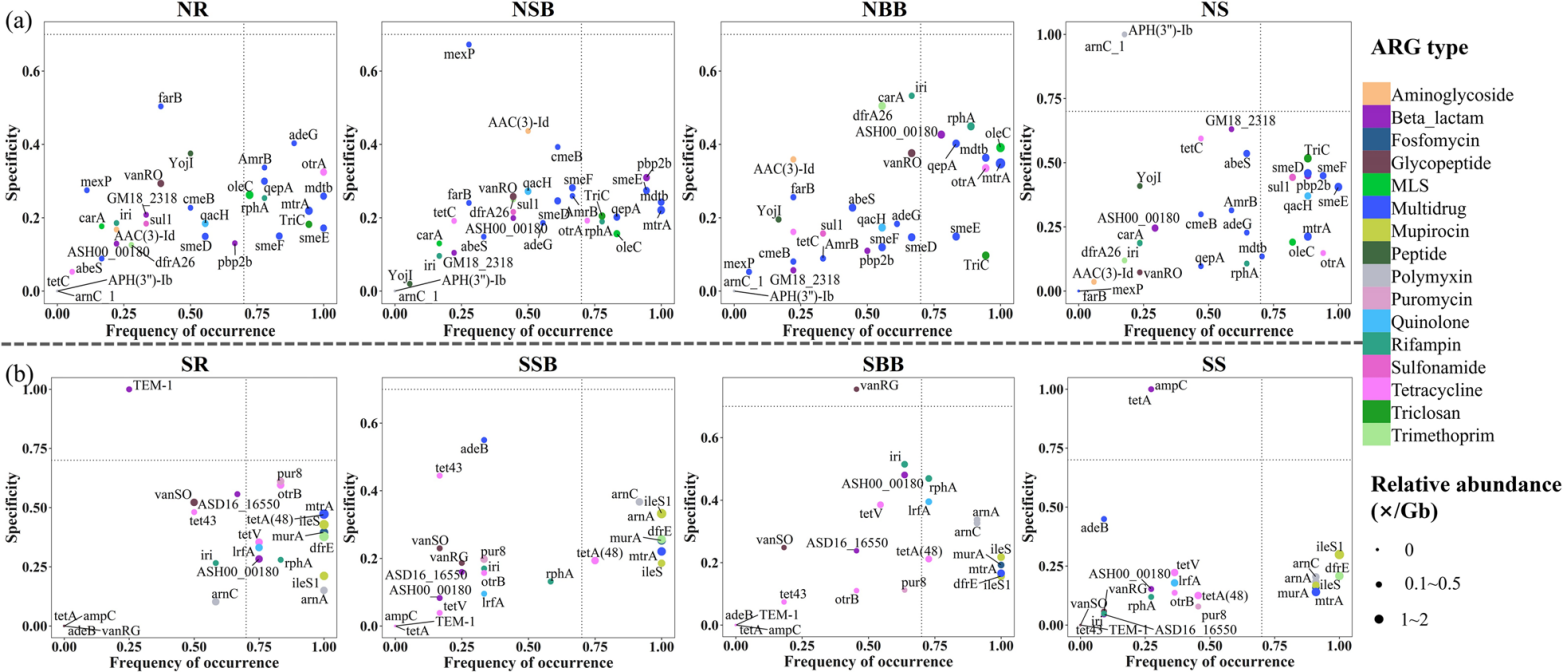
SPEC-OCCU plots show ARGs that differ between the four habitats in the south or north of China. **a** NR versus NSB versus NBB versus NS; **b** SR versus SSB versus SBB versus SS; the x-axis represents occupancy, i.e. how well an ARG is distributed across all sites of each group; and the y-axis represents specificity, i.e. whether ARGs are also found in other groups. (For abbreviations, see legend of Fig. 2)

ARGs with significant differences between/among habitats (hereinafter referred to as “differential ARGs”) were selected using the Kruskal-Wallis test. To examine how differential ARGs are distributed across sampling sites of the same habitat and how specific they are to a habitat, the occupancy and specificity of individual ARGs were calculated for each habitat and projected onto maps (Figs. 4 and 5). Screening of differential ARGs of the same habitat in the north and south (i.e., SR vs NR, SSB vs NSB, SBB vs. NBB, and SS vs. NS) revealed that multidrug resistance genes contributed most to the north-south differentiation for each habitat (Fig. 4). Selected multidrug resistance genes of each habitat displayed a variable occupancy, but they were common in most sites of the north. Meanwhile, overall differential ARGs were more frequent and prevalent in the northern habitats. These differences in ARGs of the same habitat between north and south were due to a combination of different occurrence frequencies and habitat selections of the differential ARGs. Also, the number of differential ARGs was less in the surface bulk soil (Fig. 4b), which may explain the non-significant difference in ARGs between SSB and NSB (Fig. 2e). Differential ARGs in the southern and northern habitats were also identified (i.e., SR vs SSB vs SBB vs. SS and NR vs. NSB vs. NBB vs. NS) (Fig. 5). In the south, tetracycline and beta-lactam resistance genes accounted for the highest proportion of differential genes. While in the north, the dominant differential genes were multidrug resistance genes. Differences in ARG profiles among the southern or northern habitats were mainly due to the different occurrence frequencies of differential ARGs.

### Co-occurrence pattern between the riverine ARGs and bacteria

**Fig. 6:**
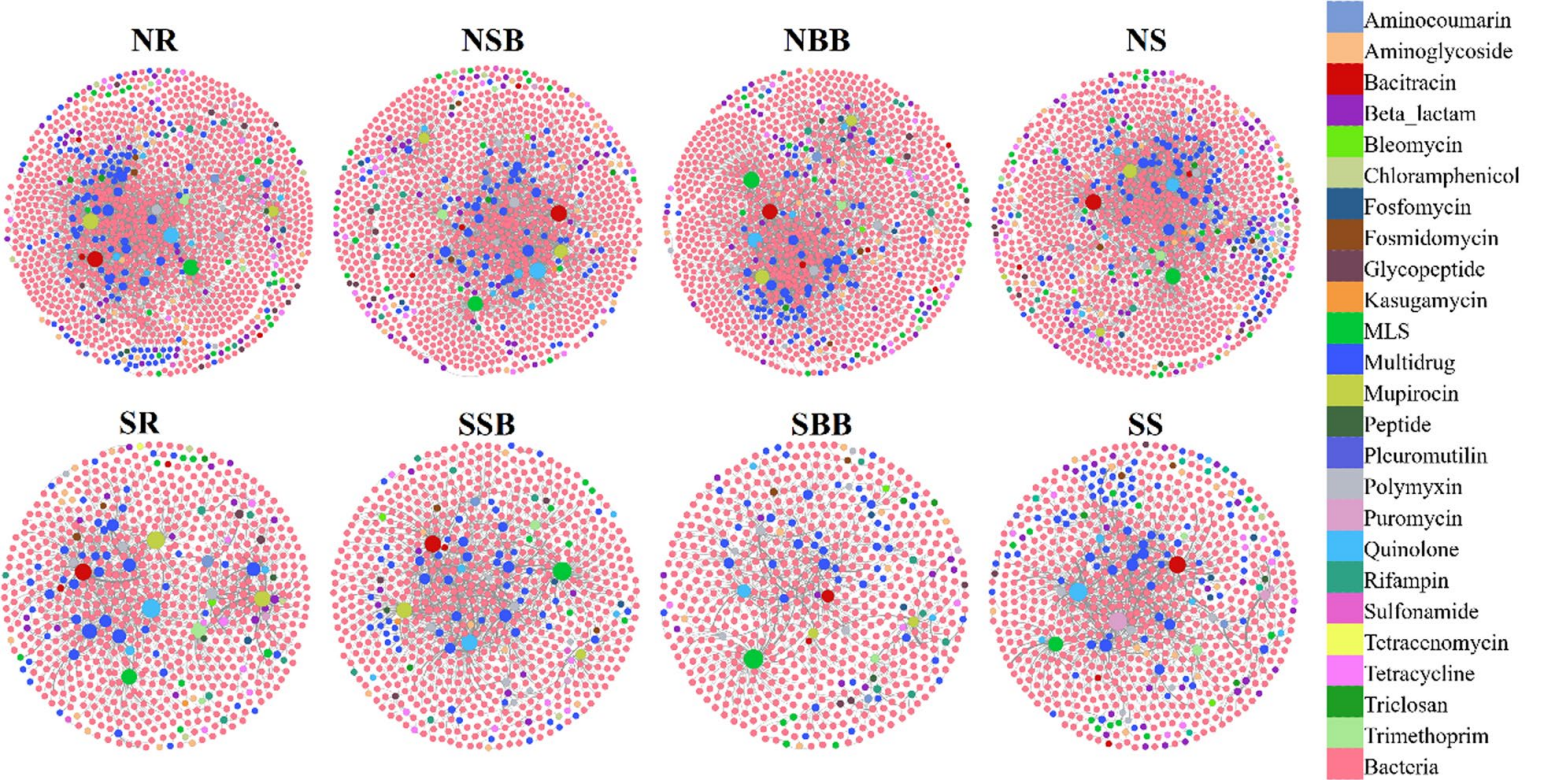
Network diagrams reveal co-occurrence of ARG subtypes and bacteria (species) on individual contigs. The presence of a linkage between ARG and bacterial species is indicated by co-occurrence on the same contig. Line size between ARG and species indicates the number of contigs that can be related to a host in a specific habitat; higher contig numbers are represented by thicker lines between the two nodes. (For abbreviations, see legend of Fig. 2)

A Mantel test revealed that the ARG profile was significantly correlated with the bacterial species in each habitat (Additional file 1: Table S3). Subsequently, the contigs carrying ARGs were subjected to taxonomic annotation. The annotation results are visualized in Fig. 6 using network diagrams. Obviously, host relationships were simpler in the southern than in the northern habitats (Additional file 1: Table S4). The most complex host relationships occurred in NR. In all eight habitats, *ile*S1, *mac*B, uppP and mfd showed a wide range of host species, which mainly belonged to the Acidobacteria, Firmicutes, Proteobacteria, and Verrucomicrobia phyla. *Massilia sp. LC238*, *Enterobacter cloacae*, *Pseudomonas stutzeri*, *Sideroxydans lithotrophicus*, and *Citrobacter sp. 30_2* were the predominant hosts of SR, NR, NSB, SBB, and NS, *Pseudomonas alcaligenes* was the predominant host of SSB SS and NBB, and all the hosts belonging to the phylum Proteobacteria. In any habitat of this study, the main hosts of core ARGs were Proteobacteria.

### Co-occurrence pattern between the riverine ARGs and MGEs

In the same way, the co-occurrence of ARGs and MGEs in the riverine ecosystems of eastern China was studied. Significant correlations between ARGs and MGEs were found in each habitat (Additional file 1: Table S5). Next, the co-occurrence of ARGs and MGEs on the assembled contigs was analysed (Additional file 1: Table S6). The analyses showed a higher degree of co-occurrence of ARGs and MGEs in the northern than in the southern habitats. Overall, the quinolone (i.e., *qac*H) and sulfonamide (i.e., *sul*1 and *sul*2) resistance genes were the most frequently co-occurring with MGEs at the contig level. Noticeably, there was a frequent co-occurrence of ARGs with *qac*Edelta. Furthermore, only one core ARG *mex*W was found to co-occur with MGEs.

### Effects of vegetation and environmental factors on ARG profiles

The results showed that there was a significant difference in ARGs between the northern and southern rhizosphere soils (*P* = 0.035). However, there was no significant difference in ARGs between the rhizosphere soils and bulk soils, neither in the south nor the north. Meanwhile, ARG abundance was not significantly correlated with vegetation richness, vegetation coverage and plant biomass (Additional file 2: Fig. S6, Additional file 1: Table S7). Further analyses revealed differential ARGs between the northern and southern rhizosphere soils, and between the bulk soils and the rhizosphere soils, and these differential ARGs showed variable occurrence frequency and specificity (Fig. 5a, Additional file 2: Fig. S7).

The grouping nesting approach was used to analyse the drivers of differences in ARG emergence, revealing the drivers (including climatic parameters, land use types, human population density, and physicochemical parameters) that lead to differences in ARGs among different habitats (Additional file 1: Table S7). The results indicated that the north-south differences in ARGs were due to a combination of multiple drivers (i.e. population density, longitude, elevation, MAP, pH, AFe, TOC, clay, cultivated land, forest land, grassland, water area, and building land). AFe was also the only factor contributing to the observed ARG differences among the four southern habitats. Elevation was a common driver of all ARG differences that occurred in this study.

**Fig. 7:**
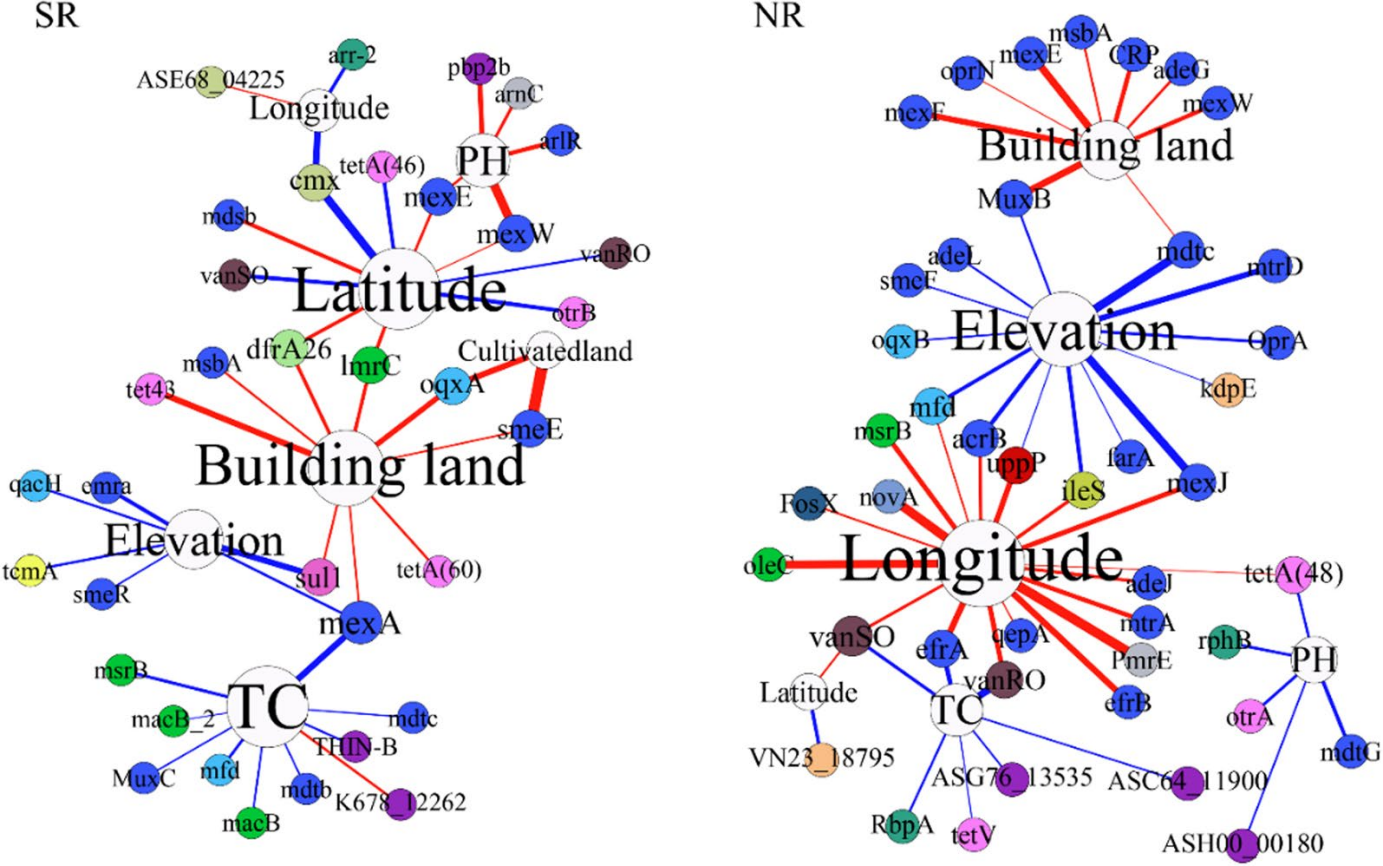
Network diagrams reveal correlations between individual ARGs and individual parameters in different habitats. Blue and red lines represent negative and positive relationships, respectively. (ρ > 0.8 or ρ < − 0.8, *P* > 0.01)

The RDA results revealed that different parameters influenced ARG profiles in the different habitats (Additional file 1: Fig. S8). The building land of all habitats, except the bottom bulk soil, showed significant correlations with ARGs. Meanwhile, ARGs were significantly correlated with latitude in all habitats except the surface bulk soil. To dissect the effects of surroundings on ARGs, this study performed correlation analyses between individual ARGs and individual parameters for each of the eight specific habitats (Fig. 7, Additional file 1: Fig. S9). The proportion of building land among all habitats, except SBB and NBB, indicated a significant positive correlation with ARGs. In contrast, other parameters (i.e. population density, latitude, longitude, elevation, cultivated land, forest land, clay, silt, sand, MAT, MAP, AFe, NH_4_^+^, pH, and TC) had both significant positive and negative correlations with ARGs in the riverine ecosystem of eastern China.

## Discussion

In the last several years, ARGs of various environments have been intensively investigated, and these genes often show very different distribution characteristics [38–41]. Yet, it is undeniable that ARGs are ubiquitously distributed in natural ecosystems [42], and ARG prevalence in the riverine ecosystems of eastern China confirms this view. In this study, twenty-four resistance gene types consisting of 469 subtypes were detected. Compared with a study in the Yarlung Tsangpo River [43], ARG subtypes in this study were more diverse, but the number of ARG subtypes in this study was lower than in the Chaobai River [44]. Variations in ARG subtype number may be related to differences in human activities, physicochemical parameters, bacterial community, MGEs, and different sample types studied [22, 45].

In this study, ARG composition and diversity characteristics were obviously different from those of unpolluted rivers, but they were similar to rivers that are subject to multiple disturbances [44, 46]. Multidrug resistance genes, MLS resistance genes, and beta-lactam resistance genes were widely found in the river ecosystems of eastern China, suggesting that there may be constant ARG sources in the studied river systems, such as direct discharge of antibiotics or ARGs with wastewater [47]. The spread of multidrug resistance genes in riverine ecosystems is alarming as it may cause microbial resistance to other and even novel antibiotics, leading to increased antibiotic failure for disease treatments [48]. The high abundance and frequency of multidrug resistance genes suggested the severity of the antibiotic resistance problem in the riverine ecosystem of eastern China. Moreover, the high abundance and frequency of beta-lactam resistance genes may be a result of the widespread use of beta-lactams in Chinese clinics [49]. Core ARGs such as *acr*B, *bep*E, *mex*F, *mex*W, *msb*A, *sav1866*, *dfr*E, *ile*S1, *mac*B, and *mfd* were the most prevalent ARGs in the riverine ecosystems of eastern China, confirming the high natural prevalence of these ten genes. The characteristics of core ARGs in this study were similar to those in the Chaobai River [46]. *Mac*B and *sav*1866 are key examples of ATP-binding cassette (ABC) transporters present in all three domains of life [50, 51]. In addition, gene *mcr*-1 can render bacteria resistant to colistin, an old antibiotic compound that is an important last-resort drug for some multidrug-resistant infections [52]. The emergence of *mcr*-1 in the riverine ecosystems of eastern China should attract attention, though *mcr*-1 abundance and frequency were very low.

South-north differences in ARG occurrence and types were observed in this study, except for surface bulk soils. Different environmental parameters, climatic conditions, and the extent of human activities seem to be responsible for differences in ARG occurrence [53, 54]. The Qinling-Huaihe Line can be regarded as an important geographical division between northern and southern China. There are pronounced south-north differences in natural conditions, geographical features, agricultural production, and people’s living customs [21], all of which could be responsible for the observed south-north differences in retrieved ARGs. In addition, the higher density of antibiotics emissions in the north than in the south may be one of the major reasons for the observed biogeographical differences in ARG profiles [22]. Investigation among urban and peri-urban river stretches showed that the unrestrained anthropogenic and related activities could potentially contribute to the overall dismal conditions and influence the connected riverine stretches on the outskirts of cities, potentially resulting in altered bacterial communities [55]. In turn, changes in bacterial communities can affect the assignment of ARGs. Unlike ARG distribution in the north, there were significant differences in ARGs among habitats in the south. Furthermore, the mutual contribution of ARGs differed among different habitats in the north vs. the south. The numerical similarity in the mutual contribution of ARGs and non-significant differences in ARGs across northern habitats demonstrate that ARGs in northern habitats may have a higher transmission rate than in the southern habitats. This difference in ARG transmission may be related to horizontal gene transfer (HGT) or microbial interactions in different habitats. Changes in bacterial communities can directly affect the fate of ARGs in the natural environment, which in turn affects other organisms.

To explore the underlying reasons for the observed differences in ARG occurrence, differential ARGs were identified in different groupings of habitats. The results reveal that differences in ARGs between the same habitats in the north vs. south were due to a combination of different occurrence frequencies and different habitat selections of differential ARGs. Thereby, differences among habitats between north and south were caused by differences in occurrence frequencies of differential ARGs. Differential ARG types of the studied habitats in the south consisted mainly of tetracycline and β-lactam resistance genes, which may be related to different sources of the two ARG types and environmental factors at the sampling sites. In comparison, differential ARG types of the studied habitats in the north consisted mainly of multidrug resistance genes, which may be related to a combination of different sources of these multidrug resistance genes, habitat-specific selection of ARGs, and differences in environmental variables of the studied habitats in the north. The high frequency co-occurrence of ARGs with MGE associated gene *qac*Edelta may also account for the high abundance of ARGs [27].

The rhizosphere has been identified as a hotspot of horizontal gene transfer for ARGs [56]. There was a significant difference in ARGs between the southern and northern rhizosphere soils. However, this study found that vegetation richness, vegetation coverage, and plant biomass did not significantly affect the formation of overall ARG profiles. Previous study has shown that plant species can affect the occurrence of ARGs by altering bacterial communities [57], which may be an important reason for the significant differences in ARGs between the northern and southern rhizosphere soils in this study. Moreover, there were differential ARGs between rhizosphere soils and bulk soils in either the southern or northern habitats. This suggests that vegetation may solely influence the assignment of differential ARGs, which vary in occurrence frequency and habitat selection.

In this study, significant correlations between ARG profiles and bacterial communities in different habitats were observed, suggesting that bacterial communities may be responsible for the occurrence of ARGs in the riverine ecosystems of eastern China. Overall, the ARG-host relationship in the north was more complex than that in the south. Moreover, ARG hosts differed in the eight habitats of this study, suggesting that ARGs spread among bacterial communities and vary from one habitat to another. Earlier studies have shown that ARGs are transferred horizontally via MGEs [6]. It was found that the co-occurrence of ARGs and MGEs was more frequent in the northern than in the southern habitats. The frequent co-occurrence of ARGs with specific bacterial communities and MGEs in the northern habitats may be responsible for the observed higher ARG abundance and transmission rates.

It has been suggested that variations in the soil/sediment resistome in different geographical locations are related to differences in vegetation, climate, and edaphic factors such as pH and soil organic matter, which will select for different microbial populations (and the ARGs they carry) and related functions [58–60]. The RDA results showed that the response of ARGs to surroundings differed between habitats. It is well known that selective or co-selective pressure introduced by human activities is the main cause of ARG enrichment in nature. In this study, the proportion of building land of SR, NR, SSB, NSB, SR, and NR were significantly and positively correlated with ARG abundance indicating that more intense human activities are associated with a higher ARG abundance. In contrast, other parameters often show variable relationships with ARGs in the different habitats suggesting that surroundings play an important role in shaping the resistome of natural environments [18, 61]. To improve health risk assessment, the relationship between surroundings and ARG profiles should be further explored.

Riverine ecosystems, an important resource of freshwater, are suffering from plenty of disturbances from anthropogenic activities. ARG profiles in different habitats tend to be characterized differently. The differences in ARG profiles are often due to differences in the habitat uniqueness and frequency of occurrence of some ARGs. The existing ARG profiles in each habitat were often influenced by their surroundings. As a densely populated area, the ecological safety of the riverine ecosystems in eastern China, and presumably elsewhere on earth is closely related to public health. This study provides an integrated and systematic exploration of the current state of ARG contamination in this highly-populated region.

## Conclusion

This study provides solid information on the occurrence and controlling factors of ARG distribution in different habitats of riverine ecosystems on a continental scale. Our results elucidate that ARGs are abundant and variable in the riverine ecosystems of eastern China and are characterized by ten core ARGs. Frequency of occurrence and habitat selection shape the resistome in the entire study area. The co-occurrence of ARGs with bacterial communities and MGEs is more frequent in the northern than in the southern habitats, and co-occurrence patterns vary from one habitat to another. Building lands constitute an important driver in shaping the ARG profile of some habitats in the riverine ecosystems of eastern China. The obtained results provide theoretical guidance to more specifically evaluate ARG pollution and develop mitigation strategies and management in eastern China river basins.

## Supplementary Information

**Additional file 1: TableS1. **The detailed information ofsampling sites. **Table S2. **The alpha diversity of ARGs in this study. **TableS3. **The Mantel test between ARGs and bacteria (species) in each habitats. **TableS4. **The feactures of the network diagram of ARGs and bacterial communitiesin different habitats. **Table S5. **The Mantel test between ARGs and MGEsin each habitats. **Table S5. **The Mantel test between ARGs and MGEs in eachhabitats. **Table S6. **Co-occurrence of ARGs and MGEs on specific contigs. **TableS7. **Grouping nesting approach to find the drivers that lead to differencesin ARGs in different habitats

**Additional file 2: Fig. S1**Number (**a**) and relative abundance (**b**) of ARGs detected inriverine ecosystems of eastern China. R represents rhizosphere soil; SBrepresents surface bulk soil (0-20 cm below bulk soil surface); BB represents bottombulk soil (40-60 cm below bulk soil surface); S represents sediment (0-20 cmbelow sediment surface); SR and NR represent rhizosphere soil in the south andnorth, respectively; SSB and NSB represent surface bulk soil (0-20 cm belowbulk soil surface) in the south and north, respectively; SBB and NBB represent bottombulk soil (40-60 cm below bulk soil surface) in the south and north,respectively; SS and NS represent sediment (0-20 cm below sediment surface) inthe south and north, respectively. MLS representmacrolides-lincosamides-streptogramines. **Fig.S2. **Proportion of detected ARGsin terms of number (**a**) and relative abundance (**b**). (Forabbreviations, see legend of Fig. S1). **Fig. S3.** Relative abundance ofcore ARGs in the riverine ecosystems of eastern China. (For abbreviations, seelegend of Fig. S1). **Fig. S3.** Relative abundance of core ARGs in the riverineecosystems of eastern China. (For abbreviations, see legend of Fig. S1). **Fig.S4.** The standard deviation of ARG abundance (**a**) and subtype number (**b**)in the riverine ecosystems of eastern China. (For abbreviations, see legend ofFig. S1). **Fig.S5.** Proportion of detected ARGs (number (**a**) and abundance (**b**)) in different habitats in the riverine ecosystem of easternChina. (For abbreviations, see legend of Fig. S1). **Fig. S6.** Influence of vegetation richness, coverage and plant biomasson ARG abundance. (For abbreviations, see legend of Fig. S1). **Fig. S7.** SPEC-OCCU plots showing ARGs that differ between two defined groups (i.e. SR vs. SSB&SBB, NR vs. NSB&NBB); the x-axis represents occupancy, i.e. how well an ARG is distributed across all sites of each group; and the y-axis represents specificity, i.e. whether ARGs are also found in other groups. (For abbreviations, see legend of Fig. S1).**Fig. S8.** RDA of ARGs and physiochemical variables controlling ARGs in different habitats (rhizosphere (R), surface bulk soil (SB), bottom bulk soil (BB), and sediment (S)). Note: *0.01 < P < 0.05, **P < 0.01. (For abbreviations, see legend of Fig. S1). **Fig. S9.** Network diagrams showing the correlation of individual ARGs and individual parameters in the different habitats. Blue and red lines represent negative and positive relationships, respectively (ρ > 0.8 or ρ < -0.8, P > 0.01). (The complement of Fig. 7). (For abbreviations, see legend of Fig. S1).

## Data Availability

All data generated during this study is available at Sequence Read Archive (SRA) under BioProject number PRJNA779832.

## Abbreviations

ARGs: Antibiotic resistance genes
MGEs: Mobile genetic elements
ORFs: Open reading frames
MAT: Mean annual temperature
MAP: Mean annual precipitation
TC: Total carbon
TOC: Organic carbon
TN: Total nitrogen
AFe: Available iron
EC: Electric conductivity
SR: Southern rhizosphere soil
SSB: Southern surface bulk soil
SBB: Southern bottom bulk soil
SS: Southern sediment
NR: Northern rhizosphere soil
NSB: Northern surface bulk soil
NBB: Northern bottom bulk soil
NS: Northern sediment
NMDS: Non-metric multidimensional scaling
ANOSIM: Analysis of similarities
ANOVA: Analyses of variance
SPEC-OCCU: Apecificity-occupancy
RDA: Redundant analyse
