# Supplementary material for: Frequency of occurrence and habitat selection shape the spatial variation in the antibiotic resistome in riverine ecosystems in eastern China
Source: Environ Microbiome. 2022 Nov 2;17:53. doi: 10.1186/s40793-022-00447-9 (PMC9632137; doi:10.1186/s40793-022-00447-9)
Supplement: Supplementary file 2 — Additional file 2: Fig. S1Number (a) and relative abundance (b) of ARGs detected inriverine ecosystems of eastern China. R represents rhizosphere soil; SBrepresents surface bulk soil (0-20 cm below bulk soil surface); BB represents bottombulk soil (40-60 cm below bulk soil surface); S represents sediment (0-20 cmbelow sediment surface); SR and NR represent rhizosphere soil in the south andnorth, respectively; SSB and NSB represent surface bulk soil (0-20 cm belowbulk soil surface) in the south and north, respectively; SBB and NBB represent bottombulk soil (40-60 cm below bulk soil surface) in the south and north,respectively; SS and NS represent sediment (0-20 cm below sediment surface) inthe south and north, respectively. MLS representmacrolides-lincosamides-streptogramines. Fig.S2. Proportion of detected ARGsin terms of number (a) and relative abundance (b). (Forabbreviations, see legend of Fig. S1). Fig. S3. Relative abundance ofcore ARGs in the riverine ecosystems of eastern China. (For abbreviations, seelegend of Fig. S1). Fig. S3. Relative abundance of core ARGs in the riverineecosystems of eastern China. (For abbreviations, see legend of Fig. S1). Fig.S4. The standard deviation of ARG abundance (a) and subtype number (b)in the riverine ecosystems of eastern China. (For abbreviations, see legend ofFig. S1). Fig.S5. Proportion of detected ARGs (number (a) and abundance (b)) in different habitats in the riverine ecosystem of easternChina. (For abbreviations, see legend of Fig. S1). Fig. S6. Influence of vegetation richness, coverage and plant biomasson ARG abundance. (For abbreviations, see legend of Fig. S1). Fig. S7. SPEC-OCCU plots showing ARGs that differ between two defined groups (i.e. SR vs. SSB&SBB, NR vs. NSB&NBB); the x-axis represents occupancy, i.e. how well an ARG is distributed across all sites of each group; and the y-axis represents specificity, i.e. whether ARGs are also found in other groups. (For abbreviations, see legend of Fig. S1).Fig. S8. [file 40793_2022_447_MOESM2_ESM.docx]

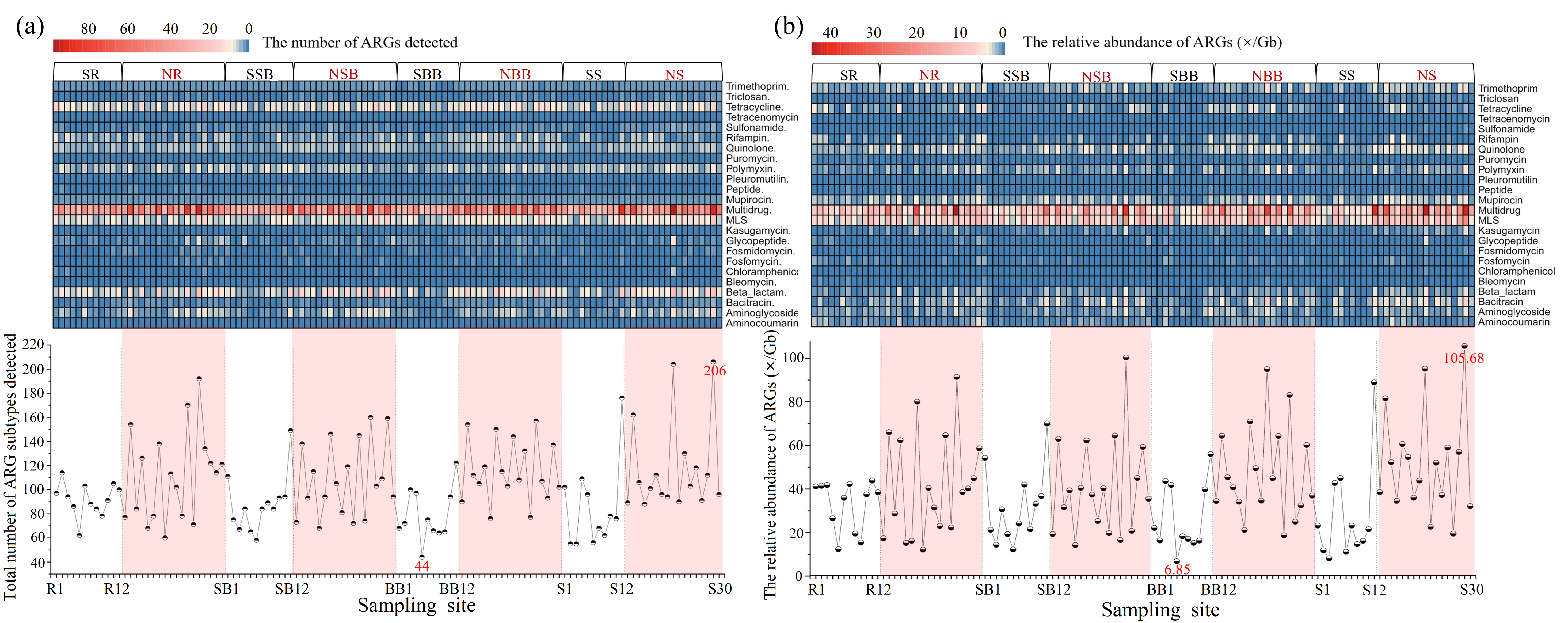


Fig. S1 Number (**a**) and relative abundance (**b**) of ARGs detected in riverine ecosystems of eastern China. R represents rhizosphere soil; SB represents surface bulk soil (0-20 cm below bulk soil surface); BB represents bottom bulk soil (40-60 cm below bulk soil surface); S represents sediment (0-20 cm below sediment surface); SR and NR represent rhizosphere soil in the south and north, respectively; SSB and NSB represent surface bulk soil (0-20 cm below bulk soil surface) in the south and north, respectively; SBB and NBB represent bottom bulk soil (40-60 cm below bulk soil surface) in the south and north, respectively; SS and NS represent sediment (0-20 cm below sediment surface) in the south and north, respectively. MLS represent macrolides-lincosamides-streptogramines.


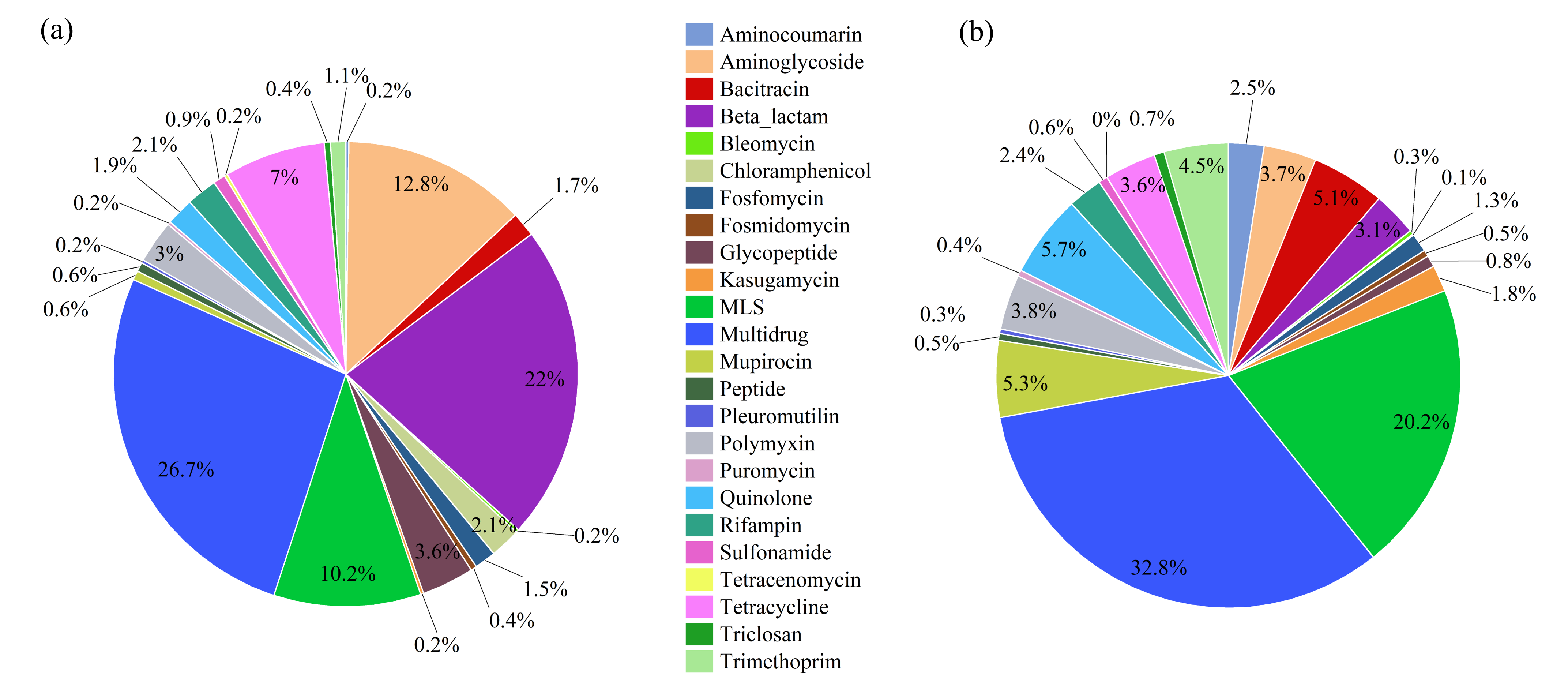


Fig. S2 Proportion of detected ARGs in terms of number (**a**) and relative abundance (**b**). (For abbreviations, see legend of Fig. S1).


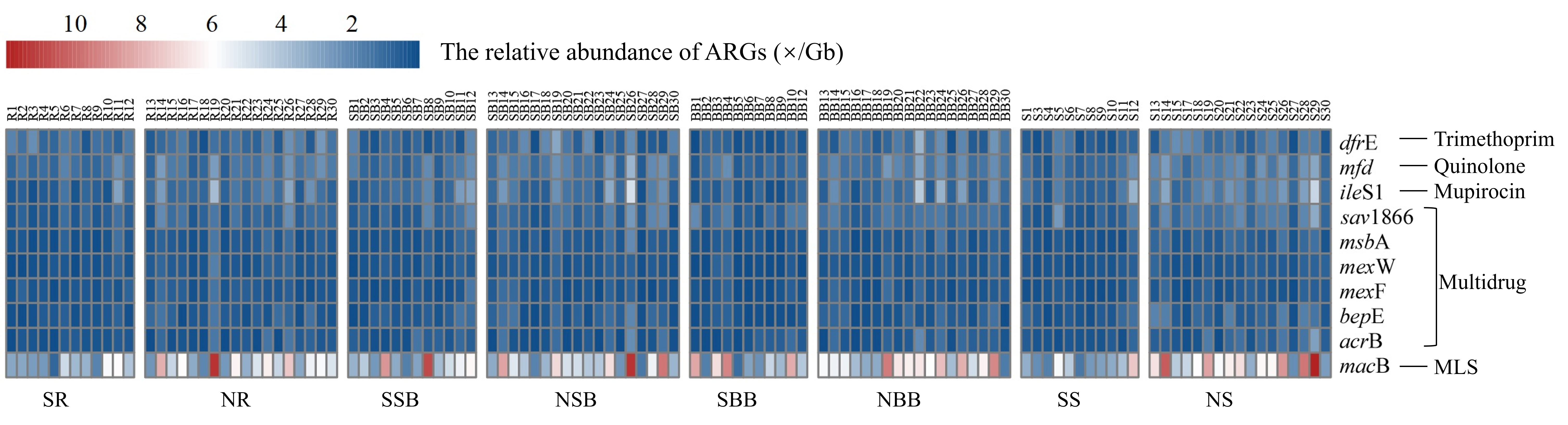


Fig. S3 Relative abundance of core ARGs in the riverine ecosystems of eastern China. (For abbreviations, see legend of Fig. S1).


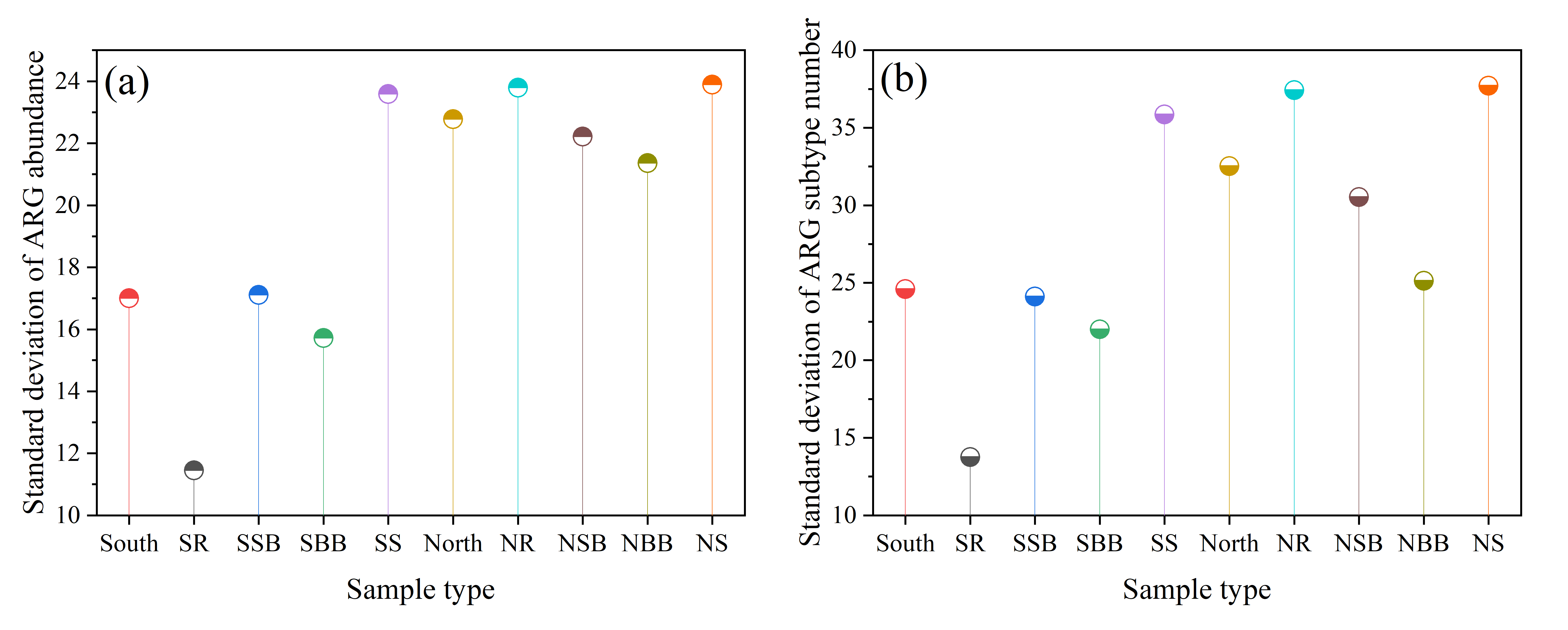


Fig. S4 The standard deviation of ARG abundance (**a**) and subtype number (**b**) in the riverine ecosystems of eastern China. (For abbreviations, see legend of Fig. S1).


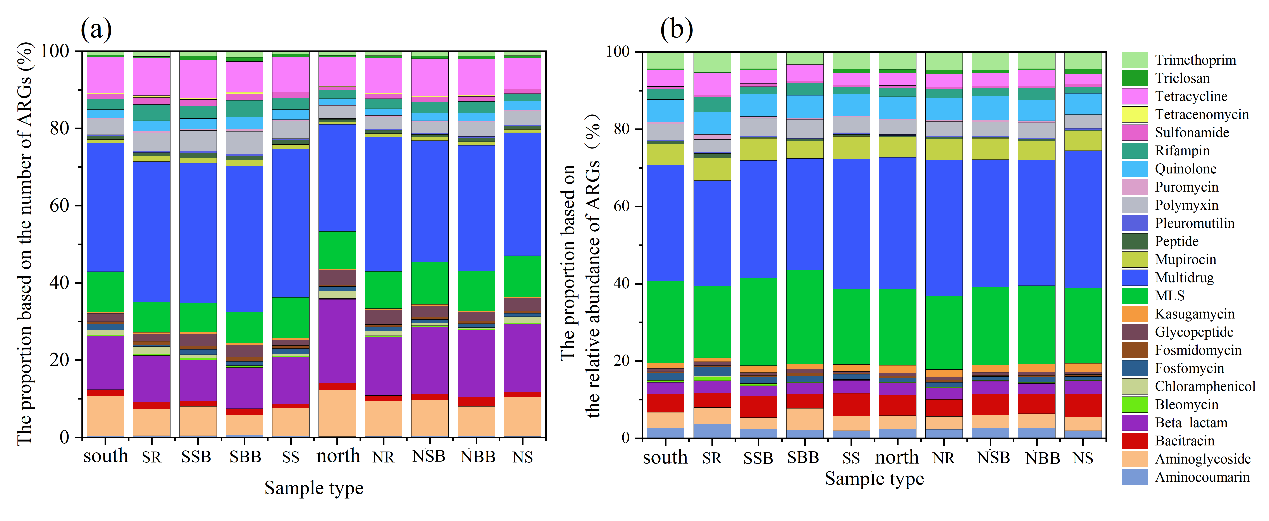


Fig. S5 Proportion of detected ARGs (number (**a**) and abundance (**b**)) in different habitats in the riverine ecosystem of eastern China. (For abbreviations, see legend of Fig. S1).


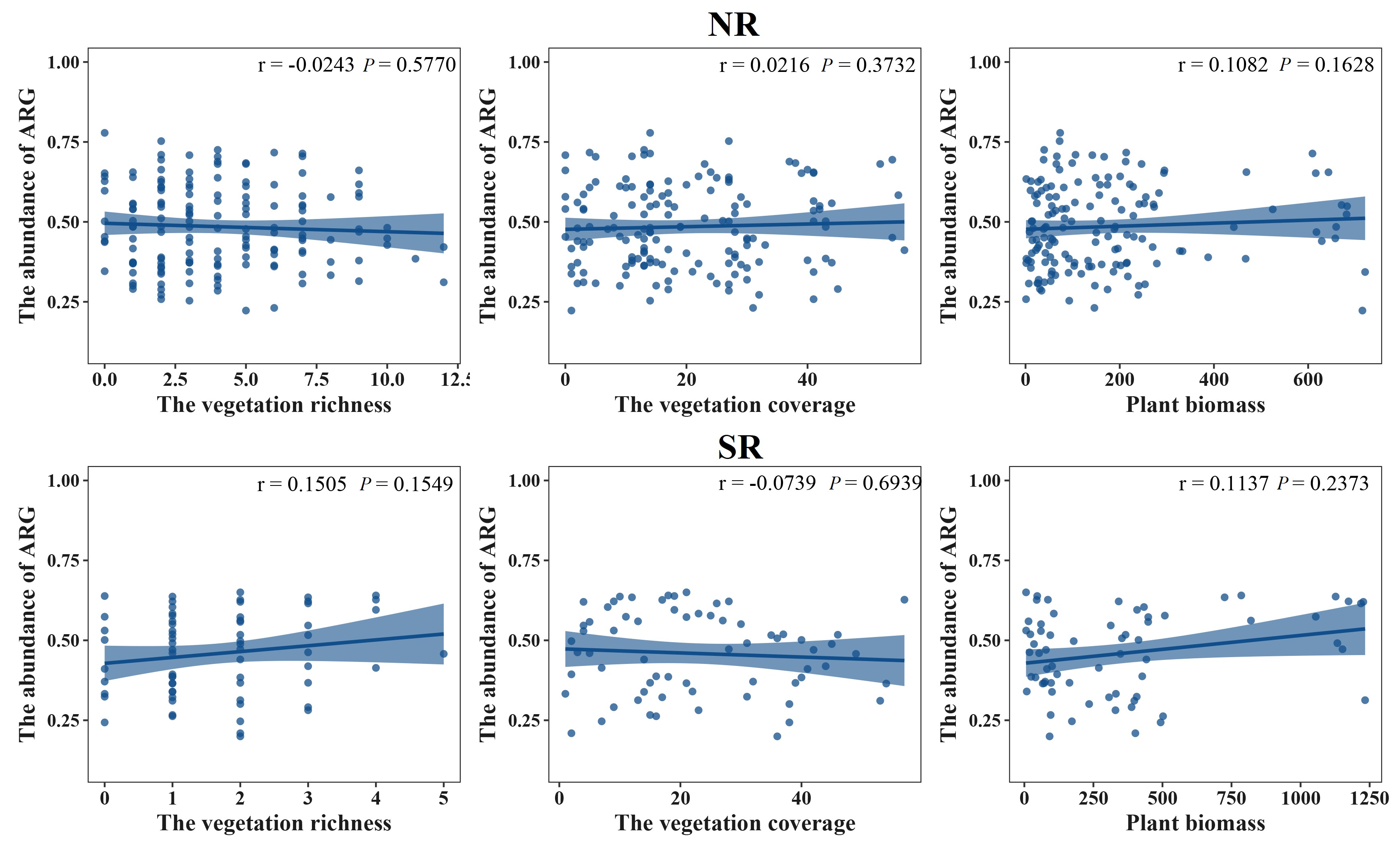


Fig. S6 Influence of vegetation richness, coverage and plant biomass on ARG abundance. (For abbreviations, see legend of Fig. S1).


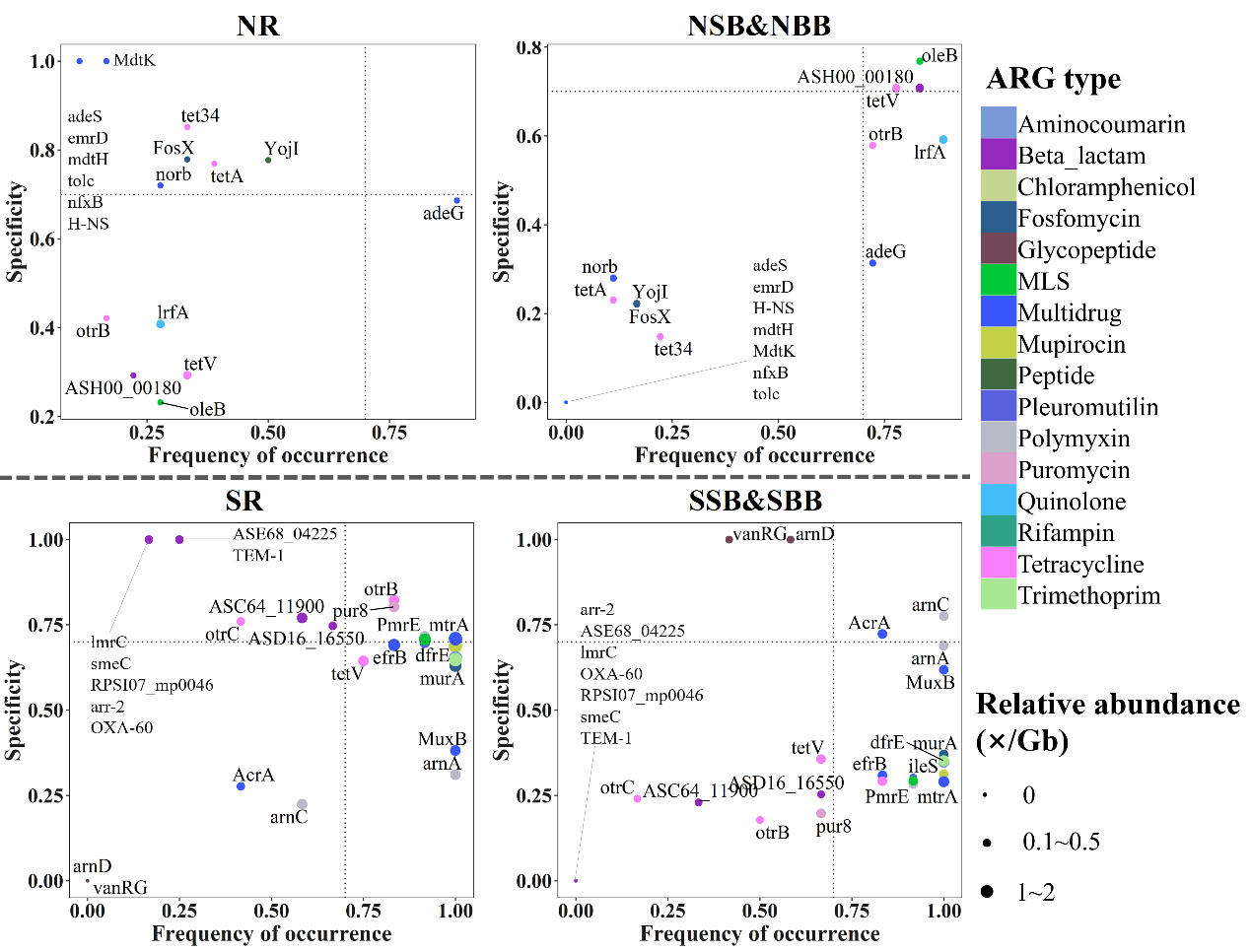


Fig. S7 SPEC-OCCU plots showing ARGs that differ between two defined groups (i.e. SR vs. SSB&SBB, NR vs. NSB&NBB); the x-axis represents occupancy, i.e. how well an ARG is distributed across all sites of each group; and the y-axis represents specificity, i.e. whether ARGs are also found in other groups. (For abbreviations, see legend of Fig. S1).


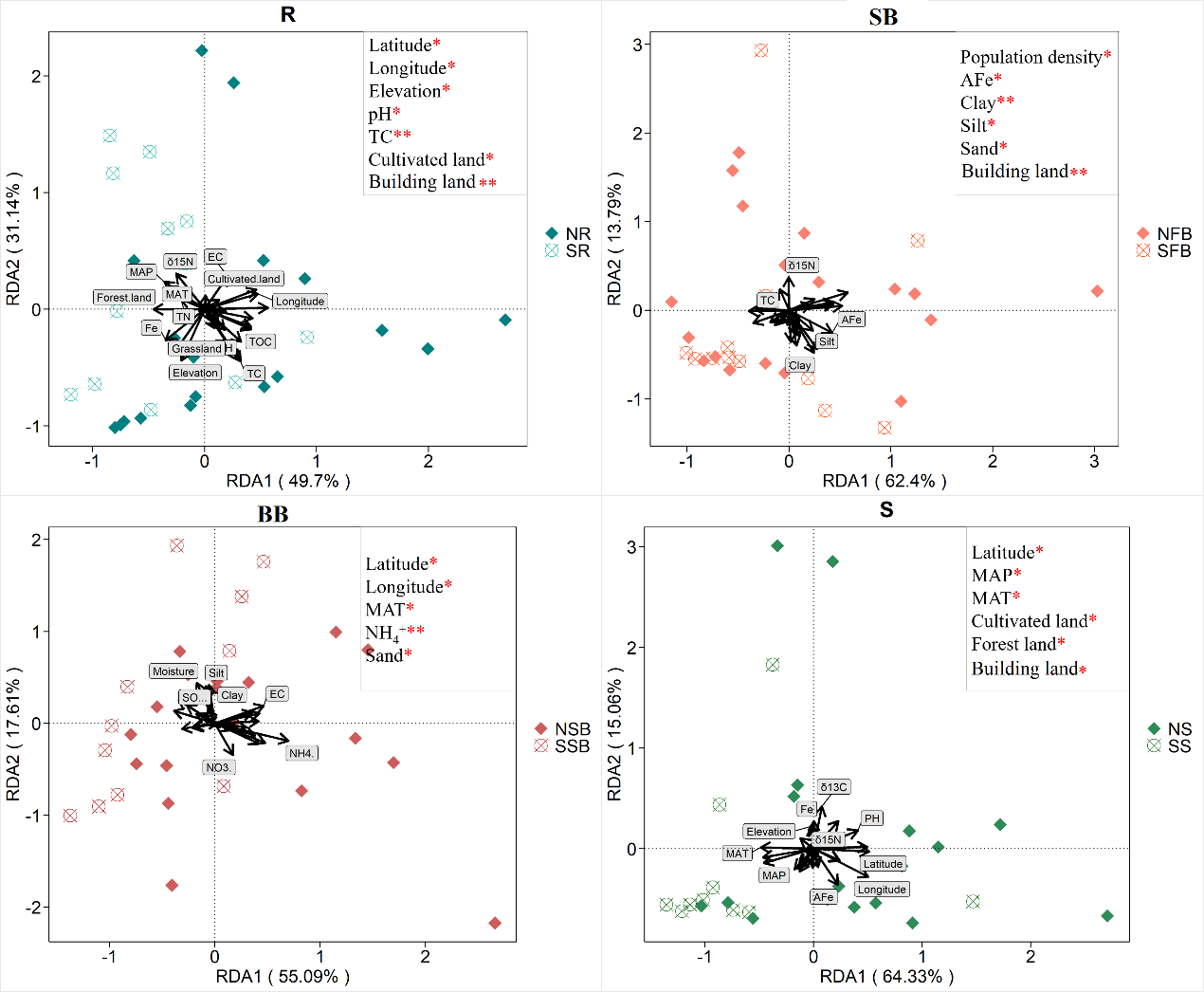


Fig. S8 RDA of ARGs and physiochemical variables controlling ARGs in different habitats (rhizosphere (R), surface bulk soil (SB), bottom bulk soil (BB), and sediment (S)). Note: *0.01 < *P* < 0.05, ***P* < 0.01. (For abbreviations, see legend of Fig. S1).


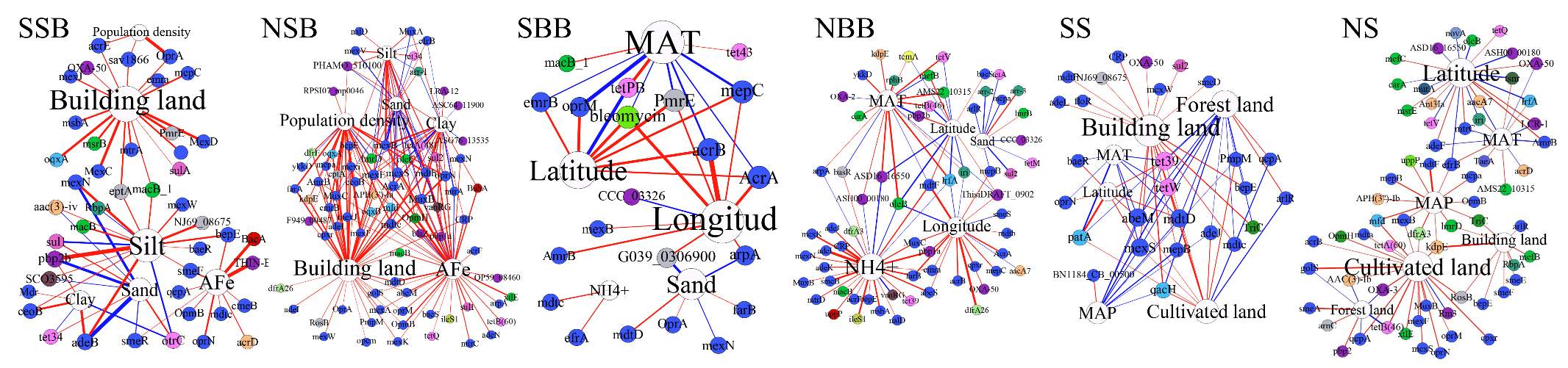


Fig. S9 Network diagrams showing the correlation of individual ARGs and individual parameters in the different habitats. Blue and red lines represent negative and positive relationships, respectively (ρ > 0.8 or ρ < -0.8, *P* > 0.01). (The complement of Fig. 7. For abbreviations, see legend of Fig. S1).
